# Capecitabine and cisplatin (XP) combination systemic chemotherapy in heavily pre-treated HER2 negative metastatic breast cancer

**DOI:** 10.1371/journal.pone.0171605

**Published:** 2017-02-24

**Authors:** Jieun Lee, Hyun Ho Kim, Sang Mi Ro, Ji Hyun Yang

**Affiliations:** 1 Division of Medical Oncology, Department of Internal Medicine, College of Medicine, The Catholic University of Korea, Seoul, Korea; 2 Cancer Research Institute, The Catholic University of Korea, Seoul, Korea; University of North Carolina at Chapel Hill School of Medicine, UNITED STATES

## Abstract

**Purpose/Objective(s):**

After taxane and anthracycline failure, no standard chemotherapy regimen is established in metastatic breast cancer (MBC). Capecitabine and cisplatin (XP) combination shows promising results in gastrointestinal cancer, but there are relatively scarce data in MBC. We reviewed the clinical outcome of XP regimen in anthracycline and taxane resistant, heavily pretreated MBC patients.

**Materials/Methods:**

Between Jan. 2010 to Feb. 2016, 48 HER2 negative MBC patients who failed anthracycline and taxane based chemotherapy were enrolled. In 43.8% of patients, more than 4 regimens were administrated before XP. Thirty-four patients (70.8%) were hormone receptor (HR) positive MBC. Patients were treated with XP (capecitabine [2000mg/m^2^ per oral; day 1–14] plus cisplatin [60mg/m^2^ IV; day 1], every 3 weeks) regimen.

**Results:**

Median progression-free survival (PFS) in total population was 4.33 months (range 1.1~33.57 months). HR positive patients showed trends for superior PFS compared to triple negative breast cancer (TNBC), without statistical significance (6.53 vs. 3.83 months, P = 0.168). In HR positive group, patients receiving 3 or less lines of chemotherapy showed superior PFS compared to others (10.1 vs. 3.0 months, P = 0.039). In multivariate analysis, HR positive patients receiving 3 or less lines of regimens still showed superior PFS (HR = 2.624, 95% CI; 1.071~6.43, P = 0.032). Most common toxicity was grade 3–4 neutropenia, without treatment-related deaths.

**Conclusions:**

XP combination regimen showed clinical benefit with tolerable toxicity in heavily pretreated patients, including HR positive patients. After anthracycline and taxane failure, early administration of XP regimen in selected patients may have improve clinical outcome in breast cancer.

## Introduction

Breast cancer is most common cancer in women worldwide [1], and second most common after thyroid cancer in Korea [2]. Although most of patients are diagnosed at early stage, 5–10% of patients are diagnosed as metastatic breast cancer (MBC) at initial presentation and up to 70% of node-positive breast cancer patients eventually relapse during follow-up [3]. In advanced breast cancer (ABC) or MBC, anthracycline or taxane-based regimens are initially used for systemic treatment [4]. However, no standard therapeutic regimen is established after anthracycline and taxane failure in ABC or MBC [5,6].

Capecitabine is an oral fluropyrimidine agent used as single agent in breast and gastrointestinal cancer patients. Combination of cisplatin with 5-FU has shown synergistic effect in prior study [7], but the clinical effect of cisplatin is not clearly analyzed in breast cancer compared to gastrointestinal cancer. Previous studies have shown the clinical efficacy of capecitabine and cisplatin (XP) combination regimen in unselected MBC patients, but with different patient population and different dosage, schedule of chemotherapeutic agents [8–10]. Considering the toxicity of cisplatin in heavily pretreated patients [9], there are relatively scarce reports about combining cisplatin to capecitabine.

In this present study, authors analyzed the clinical efficacy and toxicity of XP combination regimen in heavily pretreated, HER2-negative breast cancer patients who shows resistance to anthracycline and taxane.

## Materials and methods

### Patients

From January 2010 to Feburary 2016, the medical records of patients who were diagnosed as recurrent or metastatic breast cancer at Seoul St. Mary’s Hospital were retrospectively reviewed. All patients were treated with capecitabine and cisplatin combination chemotherapy after progression of anthracycline and taxane treatment at the time of study enrollment. Forty-eight patients who fulfilled prior criteria were enrolled for analysis. The other eligible criteria were as follows; (1) pathologically proven invasive ductal or lobular carcinoma by surgical or biopsy specimen; (2) an Eastern Cooperative Oncology Group (ECOG) performance status of 0 to 2; (3) evaluable lesion based on Response Evaluation Criteria in Solid Tumors (RECIST) ver. 1.1; (4) adequate bone marrow function, renal function, and hepatic function. Patients with gastrointestinal obstruction were excluded from the study. This study was approved by the Institutional Review Board (IRB) of Seoul St. Mary’s Hospital, Catholic University of Korea.

### Treatment schedule and response evaluation

Patients were treated with the combination of capecitabine (1000mg/m^2^ twice a day, oral administration; 14 days of treatment followed by 7 days of rest) and cisplatin (60mg/m^2^, intravenous [IV]; day 1) (XP) every 3 weeks. One liter of half saline was delivered before and after administration of cisplatin. Response evaluation was performed based on CT scans every 2 cycles of chemotherapy, using Response Evaluation Criteria in Solid Tumors (RECIST) criteria ver. 1.1. Toxicity was assessed based on National Cancer Institute Common Terminology Criteria for Adverse Events, ver. 4.0, during each cycles of chemotherapy. Chemotherapy was administered until progressive disease or unacceptable toxicity was observed. Chemotherapy was suspended if patient showed intolerance to chemotherapy.

### Statistical analysis

Overall survival (OS) was calculated from the start date of first XP chemotherapy to patients’ death or last follow-up date. Progression free survival (PFS) was calculated from the start date of XP chemotherapy to the date of cancer progression, proven by CT scans. The disease control rate (DCR) was defined as patient proportion showing partial response (PR) of stable disease (SD) based on RECIST criteria. OS and PFS were analyzed using log-rank test and Kaplan-Meier method. Cox regression analysis was done to analyze the relationship between the clinicopathologic prognostic factors and OS, PFS. All statistical analyses were carried out using SPSS, version 24.

## Results

### Patient characteristics

Between Jan 2010 to Feb 2016, 48 recurrent or metastatic breast cancer patients who showed progression after anthracycline and taxane administration were enrolled for the study. Baseline patient characteristics are described in Table 1. The median age of patient population was 51 years. Two patients were diagnosed at their thirties, and rest of the patients was more than 40 years of age. There was no familial history for breast cancer or ovary cancer. Forty-one patient showed recurrent breast cancer. Among recurrent breast cancer patients, 26 patients (63.4%) received anthracycline and taxane as neoadjuvant or adjuvant chemotherapy. Thirty-two patients (66.7%) received 3 or more lines of systemic chemotherapy at palliative setting, which shows more than half of the patients were heavily pretreated. Among total patient population, 34 patients (70.8%) were diagnosed as hormone receptor (HR) positive breast cancer. Twenty patients (58.8%) among HR positive group were pretreated with aromatase inhibitor (AI). Twenty-one patients (43.8%) were pretreated with cisplatin-containing doublet chemotherapy regimen before administration of XP.

**Table 1:** Characteristics of patient population.

	No. (%)
No. of patients	48
Age (years)	
Median	51
Range	31~70
ECOG	
0	11 (22.9)
1	28 (58.3)
2	9 (18.8)
Hormone receptor positive	34 (70.8)
Previous exposure of AI^*^	20 (58.8)
Triple negative breast cancer	14 (29.2)
Recurrent breast cancer	41 (85.4)
Previous neoadjuvant or adjuvant anthracycline and taxane	26 (63.4)
Initial stage IV breast cancer	7 (14.6)
Previous lines of palliative chemotherapy	
First	4 (8.3)
Second	12 (25)
Third	11 (22.9)
≥ Fourth	21 (43.8)
Previous chemotherapy exposure	
Anthracycline and Taxane	48 (100)
Gemcitabine	23 (47.9)
Cyclophosphamide	6 (12.5)
Vinorelbine	8 (16.7)
Eribulin	4 (8.3)
Cisplatin combination doublet	21 (43.8)
Metastatic sites	
Brain	5 (10.4)
Bone	23 (47.9)
Lung	19 (39.6)
Liver	13 (27.1)
Skin & Soft tissue	5 (10.4)
Pericardium	3 (6.3)
Chest wall	8 (16.7)
Lymph node	15 (31.3)

^*^ AI: Aromatase Inhibitor.

### Treatment response

Total 181 cycles of XP chemotherapy was delivered to 48 patients. Median 4 cycles of XP chemotherapy was administered per patient. The average relative dose intensity was 81 ± 8.79% for capecitabine and 81.3 ± 8.53% for cisplatin, respectively. In HR positive group, PR was achieved in 12 patients (35.3%) and SD in 13 patients (38.2%), resulting 73.5% of DCR. In triple-negative breast cancer (TNBC) group, PR was detected in 3 patients (21.4%) and SD in 6 patients (42.9%), with 64.3% of DCR (Table 2).

**Table 2:** Clinical outcomes.

	HR positive	TNBC
Response		
^*^PR	12 (35.3%)	3 (21.4%)
^**^SD	13 (38.2%)	6 (42.9%)
Disease control rate (PR + SD)	25 (73.5%)	9 (64.3%)
Survival outcome		
median ^†^PFS	6.53 months	3.68 months
	(range 1.1~33.77 months)	(range 1.5 ~ 14.3 months)
median ^‡^OS in metastatic setting	11.13 months	13.79 months
	(range 2.77 ~ 48.1 months)	(range 3.23 ~ 47.4 months)

^*^ PR (partial response); ≥30% decrease in the sum of the longest diameters of target lesions.

^**^ SD (stable disease); neither PR or progressive disease.

Progressive disease; > 20% increase in the sum of diameters from nadir and an absolute increase of > 5mm

^†^PFS; Progression Free Survival.

^‡^OS; Overall Survival.

### Survival outcomes

**Fig 1:**
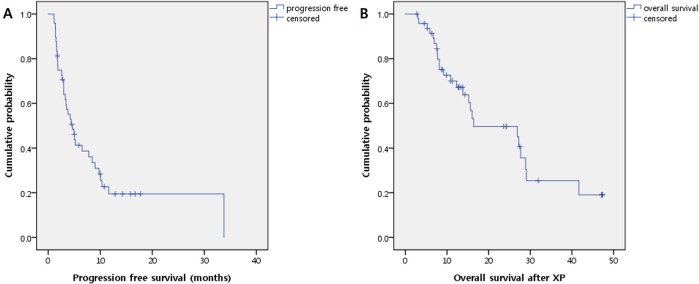
The median progression free survival (A) and overall survival (B) in total patient population.

**Fig 2:**
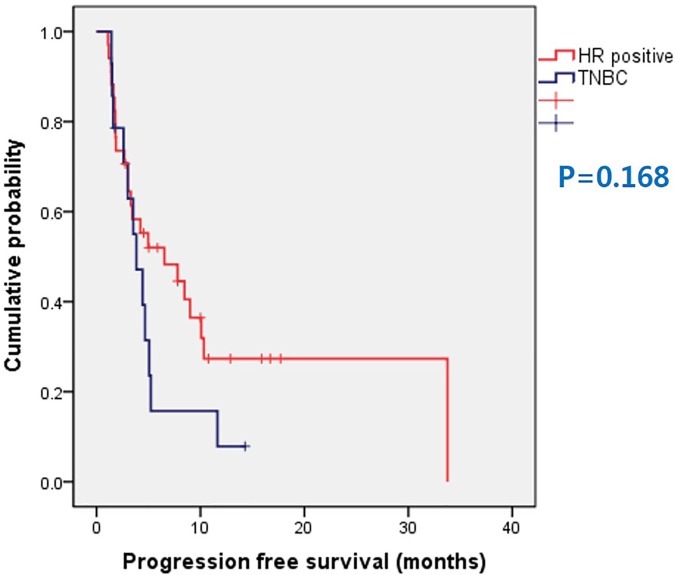
Progression free survival according to hormone receptor status.

**Fig 3:**
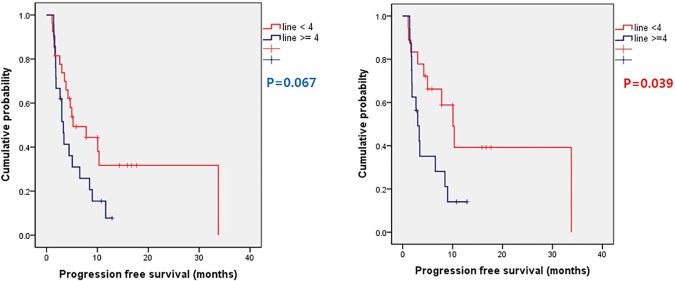
Progression free survival according to prior numbers of systemic chemotherapy in total patient population (A) and in hormone receptor positive patients (B).

The median PFS and OS of total patient population was 4.33 months (range 1.1~33.57 months) and 12.5 months (range 2.77~48.1 months) (Fig 1A and 1B). Median PFS of HR positive group was 6.53 months (range 1.1~33.77 months) and TNBC group was 3.68 months (range 1.5~14.3 months), without statistical significance (P = 0.168) (Fig 2). There were no statistical difference in OS between HR positive group and TNBC group (median OS 11.13 months vs. 13.79 months). Patients who received less than fourth line of systemic chemotherapy before XP regimen showed superior PFS compared to patients receiving fourth or more lines of chemotherapy, with borderline statistical significance (median 10.1 months vs. 3.0 months, P = 0.067) (Fig 3A). However, in HR positive patients who received less than fourth line of systemic chemotherapy showed statistically superior PFS compared to patients receiving fourth or more lines of systemic chemotherapy before XP combination regimen (median 10.1 months vs. 3.0 months, P = 0.039) (Fig 3B). There were no statistical differences of OS according to previous regimen number.

### Association of clinical factors and survival outcomes in HR positive patients

Among HR positive patients, univariate analysis was done for evaluation of relationship between clinical factors and PFS. Numbers of chemotherapy prior to XP were statistically associated to superior PFS (P = 0.045). Authors included following clinical factors for multivariate analysis; lines of chemotherapy prior to XP, prior exposure to aromatase inhibitor. Multivariate analysis still showed prior numbers of chemotherapy before XP statistically associated to better PFS (hazard ratio [HR] = 3.138; 95% CI = 1.04~9.51; P = 0.043) (Table 3).

**Table 3:** Univariate and multivariate analysis of progression free survival in HR positive patients.

	Univariate		Multivariate		
	Hazard ratio	P value	Hazard ratio	95% CI	P value
Chemotherapy lines					
lines < 4 vs. ≥4	2.414	0.045	3.138	1.04–9.51	0.043
^*^ AI_exposure					
No vs. Yes	1.447	0.407	0.742	0.25–2.19	0.590

^*^ AI: Aromatase Inhibitor.

### Toxicity profiles

During administration of systemic chemotherapy, there were no treatment-related deaths. The hematologic, non-hematologic toxicities are summarized at Table 4. The most common hematologic toxicity was grade 3–4 neutropenia (37%). Grade 4 neutropenia developed in 9 cases (5%), but there were no febrile neutropenia. The most common non-hematologic toxicity was grade 1–2 peripheral neuropathy (27.1%). There were grade 1–2 hand-foot syndrome developed in 33 cases (18.2%). All of these toxicities were manageable.

**Table 4:** Chemotherapy toxicities.

	Grade 1–2	Grade 3–4	Grade 4
Hematologic			
Anemia	30 (16.6)	2 (1.1)	0
Neutropenia	63 (34.8)	67 (37)	9 (5)
Thrombocytopenia	44 (24.3)	7 (3.9)	0
Non-hematologic			
Hand-foot syndrome	33 (18.2)	2 (1.1)	0
Peripheral neuropathy	49 (27.1)	9 (5)	0
Diarrhea	2 (1.1)	1 (0.5)	0
Nausea	12 (6.6)	1 (0.5)	0

Total 181 cycles of chemotherapy was delivered.

## Discussion

After anthracycline and taxane failure, there is no established chemotherapy regimen for ABC or MBC patients. Various cytotoxic agents including antimetabolites (capecitabine, gemcitabine), antitubulins (eribulin, vinorelbine), and platinum analogues (cisplatin, carboplatin) are used as single agent or as combination regimen after anthracycline and taxane failure [4].

Capecitabine is an oral fluoropyridine derivate used widely as single agent in breast cancer. However, capecitabine is also used as combination regimen with other chemotherapeutic agents [11–14], and some literatures report superior outcomes of combination regimen compared to capecitabine monotherapy [11,12]. Cisplatin is used in various types of cancers, and known to have synergistic effect when combined to capecitabine [7]. Capecitabine and cisplatin (XP) combination regimen is widely used in gastrointestinal cancer, but there are relatively scarce data in breast cancer patients with different administration schedule and different patient population.

In this analysis, XP combination regimen was administered to heavily pre-treated patients. About 40% of patients received 4 or more lines of systemic chemotherapy prior to XP administration. Although patients were very heavily pre-treated, clinical and survival outcomes were relatively comparable to previous literatures which included cisplatin doublet chemotherapy [8–10,15,16]. The pathologic subtype, numbers of previous chemotherapy regimen in previous studies were different compared to current study. Li et al. [8] analyzed TNBC patients, and most of the patients received XP as first line for palliative chemotherapy regimen. Other two studies [9,10] comprised breast cancer patients irrespectively to hormone status and HER2 status, and clinical and survival outcomes were relatively comparable to our analysis. In our analysis, HR positive patients showed higher DCR compared to TNBC patients, but there were no statistical difference (73.5% vs. 64.3%, P = 0.522) estimated by Pearson Chi-square test. There were trends for superior PFS in HR positive patients, but also without statistical significance. Ozdemir et al. [16] analyzed HER2 negative breast cancer population similar to our study. However, the patients in our study was more heavily pre-treated before XP. In our analysis, more than half of patients received XP as third or more lines of palliative chemotherapy, and 43.8% of patients were pre-exposed to cisplatin doublet before XP (Table 5).

**Table 5:** Previous studies of XP regimen in metastatic breast cancer patients previously treated with anthracycline and taxane.

Reference	N	subtype	Regimen	palliative line	DCR (%)	PFS (months)	OS (months)
			X: 2000mg/m^2^	1^st^: 84.8%			
Li et al. [8]	33	^*^TNBC	P: 75mg/m^2^	2^nd^: 12.1%	84.8	8.2	17.8
				3^rd^: 3.0%			
		HER2 positive	X: 2000mg/m^2^	1^st^: 25.6%			
Donadio et al. [9]	39	^**^ER positive	P: 20mg/m^2^	2^nd^: 69.3%	40.9	5.2	10.9
		TNBC	every week for 6 weeks	3^rd^: 5.1%			
		HER2 positive	X: 2000mg/m^2^	1^st^: 39.4%			
Öksüzoglu et al. [10]	33	ER positive	P: 60mg/m^2^	2^nd^: 42.4%	81.8	6.3	11.5
		TNBC		≥NB^rd^: 18.2%			
			X: 2000mg/m^2^	1^st^: 50%			
Ozdemir et al. [16]	64	HER2 negative	P: 60mg/m^2^	2^nd^: 37.5%	81.3	7	17
				3^rd^: 12.5%			
			X: 2000mg/m^2^	1^st^: 8.3%			
current study	48	HER2 negative	P: 60mg/m^2^	2^nd^: 25%	70.8	4.3	12.5
				≥2.^rd^: 66.7%			

^*^TNBC; Triple negative breast cancer.

^**^ER; Estrogen receptor.

Cisplatin showed improvement of pathologic complete response rate in neoadjuvant setting [17], and there are reports of positive benefit of cisplatin in metastatic setting [18]. But there are also conflicting reports about the role of cisplatin in TNBC patients during systemic chemotherapy [19]. Authors supposed although cisplatin has clinical benefit for TNBC, the anti-tumor effect of cisplatin could not overcome the natural poor prognosis of TNBC compared to HR positive breast cancer patients. This hypothesis might explain the clinical and survival outcomes of our analysis. However, the number of TNBC patients in our analysis is very small, and patients were not randomized in our analysis. This bias may have influenced the survival outcomes in our analysis, and further randomized study concerning the role of platinum agents in TNBC is warranted.

Considering patient population was very heavily pre-treated, authors analyzed the survival outcome according to pre-treated chemotherapy regimen numbers. Among total patient population, patients who received less than 4 lines of systemic chemotherapy showed superior PFS with borderline statistical significance (median PFS 10.1 months vs 3.0 months, respectively, P = 0.067). Subgroup analysis showed HR positive patients who received less than 4 lines of chemotherapy showed superior PFS compared to patients receiving 4 or more lines of systemic chemotherapy (median PFS 10.1 months vs. 3.0 months, respectively, P = 0.039), and multivariate analysis still showed the statistical difference according to prior lines of chemotherapy influencing PFS. In TNBC patients, there were no statistical differences of PFS according to numbers of pre-treated regimens. Unlike other solid cancers, platinum agents show markedly decreased activity when used as salvage regimen in breast cancer [20,21]. Progressive resistance of cisplatin in breast cancer may explain the difference of PFS according to pre-treated chemotherapy regimen.

In our study, XP regimen was well tolerated. Considering patients were heavily pre-treated, bone marrow suppression was the main concern during the treatment. Grade 3–4 neutropenia occurred in 37% of total delivered chemotherapy, but the toxicity was manageable with granulocyte-colony stimulating factor (G-CSF) injection and supportive care, with no treatment-related death. Anemia and thrombocytopenia was detected during chemotherapy, with grade 1–2 toxicity. Common non-hematologic toxicity was peripheral neuropathy and hand-foot syndrome. Grade 3–4 peripheral neuropathies were detected in 5% of total delivered chemotherapy cycles, and most of reported peripheral neuropathy was graded as grade 1–2. Considering all patients were previously treated with docetaxel, neuropathy was easily manageable.

This study showed there might be a potential clinical benefit of XP regimen in heavily pre-treated breast cancer patients with favorable toxicity profile. After anthracycline and taxane failure, early introduction of XP combination regimen could be considered in selected patient population, including HR positive MBC patients.

However, there are some limitations in our analysis. This analysis was conducted in retrospective manner with relatively small sample size, requiring careful interpretation of the study results. In addition, considering the incidence of TNBC is lower compared to HR positive MBC, the included number of TNBC patients were small compared to total patient population. This might have influenced the results, which might have been associated to the selection bias. However, considering there are no standard treatment guideline after anthracycline and taxane failure, heterogeneous treatment options might have dispersed the patient population who are treated with palliative chemotherapy regimen afterwards. This may have influenced to the relatively small patient number who were treated with XP in our study. However, this analysis comprised the total patients who were treated with XP regimen in single tertiary center, and was planned as a pilot study to provide a basis of multicenter clinical study to analyze the clinical benefit of XP regimen in heavily pre-treated breast cancer patients in Korea.

In conclusion, this study showed that XP combination regimen might be one of an option for patients who showed progression after anthracycline and taxane administration. XP regimen also may be considered in HR positive MBC patients, other than TNBC patients. Further large, randomized prospective clinical trial is warranted for the confirmation of this study.
